# Week-One Anaemia was Associated with Increased One-Year Mortality in Critically Ill Surgical Patients

**DOI:** 10.1155/2022/8121611

**Published:** 2022-09-06

**Authors:** Feng-Hsu Wu, Li-Ting Wong, Chieh-Liang Wu, Wen-Cheng Chao

**Affiliations:** ^1^Department of Critical Care Medicine, Taichung Veterans General Hospital, Taichung, Taiwan; ^2^Division of General Surgery, Department of Surgery, Taichung Veterans General Hospital, Taichung, Taiwan; ^3^Department of Nursing, Hung Kuang University, Taichung, Taiwan; ^4^Department of Medical Research, Taichung Veterans General Hospital, Taichung, Taiwan; ^5^Department of Post-Baccalaureate Medicine, College of Medicine, National Chung Hsing University, Taichun, Taiwan; ^6^Department of Industrial Engineering and Enterprise Information, Tunghai University, Taichung, Taiwan; ^7^Artificial Intelligence Studio, Taichung Veterans General Hospital, Taichung, Taiwan; ^8^Department of Automatic Control Engineering, Feng Chia University, Taichung, Taiwan; ^9^Big Data Center, Chung Hsing University, Taichung, Taiwan

## Abstract

**Background:**

Anaemia has a deleterious effect on surgical patients, but the long-term impact of anaemia in critically ill surgical patients remains unclear.

**Methods:**

We enrolled consecutive patients who were admitted to surgical intensive care units (ICUs) at a tertiary referral centre in central Taiwan between 2015 and 2020. We used both Cox proportional hazards analysis and propensity score-based analyses, including propensity score matching (PSM), inverse probability of treatment weighting (IPTW), and covariate balancing propensity score (CBPS) to determine hazard ratios (HRs) and 95% confidence intervals (CIs) for one-year mortality.

**Results:**

A total of 7,623 critically ill surgical patients were enrolled, and 29.9% (2,280/7,623) of them had week-one anaemia (haemoglobin <10 g/dL). We found that anaemia was independently associated with an increased risk of one-year mortality after adjustment for relevant covariates (aHR, 1.170; 95% CI, 1.045–1.310). We further identified a consistent strength of association between anaemia and one-year mortality in propensity score-based analyses, with the adjusted HRs in the PSM, IPTW, and CBPS were 1.164 (95% CI 1.025–1.322), 1.179 (95% CI 1.030–1.348), and 1.181 (1.034–1.349), respectively.

**Conclusions:**

We identified the impact on one-year mortality of anaemia in critically ill surgical patients, and more studies are needed to validate our findings.

## 1. Introduction

Anaemia is a highly prevalent comorbidity in critically ill surgical patients, and it is estimated that approximately 50% of critically ill patients experience anaemia. [1–4] A number of evidence have shown that anaemia has a deleterious impact on the outcome in patients undergoing surgery. [5–7] Baron et al. conducted a multicentre study with 39,309 patients undergoing in-patient surgery in 28 European nations and implicated anaemia with poor postsurgery outcomes, including hospital length of stay, postoperative admission to intensive care, and in-hospital mortality [5]. Similarly, Musallam et al. analysed data of 211 hospitals worldwide with 227,425 patients and found that mild anaemia, defined by haematocrit concentration <39%, (1.41, 95% CI 1.30–1.53) and moderate-to-severe anaemia, defined by haematocrit concentration <30%, (1.44, 95% CI 1.29–1.60) were associated with an increased risk of 30-day morbidity in patients undergoing major noncardiac surgery [6].

In addition to the aforementioned evidence among in-patient surgical patients, Hammer et al. focused on critically ill surgical patients and investigated factors associated with being readmitted to the intensive care units (ICUs) among 7,126 critically ill surgical patients at Beth Israel Deaconess Medical Center between 2015 and 2019. [7] Hammer et al. reported that severe anaemia (haemoglobin <7 mg/dl) was a crucial risk factor of being readmitted to ICU during the same hospitalisation. [7] Our recently published study, using an explainable machine learning approach to establish a long-term mortality prediction model in critically ill patients, found that levels of haemoglobin lower than 10 g/dL was a crucial feature with high feature importance in the long-term mortality prediction model [8]. Therefore, there is an essential need to address the long-term mortality impact of anaemia in critically surgical ill patients. In the present study, we linked the critical care database at a tertiary referral centre in central Taiwan with the population-based death-registry file in Taiwan to address the prevalence of anaemia and to investigate the long-term mortality association of anaemia in critically ill surgical patients.

## 2. Materials and Methods

### 2.1. Ethical Approval

The retrospective study was conducted in accordance with the Declaration of Helsinki and approved by the Institutional Review Board of the Taichung Veterans General Hospital (TCVGH: SE21098B). The informed consent was waived due to all of the data analysed were deidentified data.

### 2.2. Study Population

This retrospective cohort study enrolled consecutive patients who were admitted to surgical ICUs at TCVGH, a tertiary referral centre with 48 surgical ICU beds in central Taiwan, between 2015 and 2020. The first ICU admission was used among patients with more than one ICU admission. We used the average level of haemoglobin among those with more than one measurement of haemoglobin, and patients without data regarding the level of haemoglobin within the first week were excluded from analyses.

### 2.3. Primary Outcome

The main outcome of interest was the one-year all-cause mortality, and we retrieved the date-of-death from the nationwide death registration profile of the National Health Insurance Research Database (NHIRD) in Taiwan [9]. Taiwan has implemented a compulsory National Health Insurance (NHI) program since 1995, with nearly 99.9% coverage of the Taiwanese population in 2019; therefore, the date-of-death among enrolled participants in the present study can be ascertained.

### 2.4. Covariates

The TCVGH critical care data warehouse was used to retrieve data with respects to demographic data, Charlson comorbidity index (CCI), Acute Physiology and Chronic Health Evaluation (APACHE) II score, presence of shock, receiving mechanical ventilation, underwent renal replacement, management including blood transfusion, and laboratory data [10]. Previous studies, including our studies, have shown the mortality association of early (day 1–3) overall fluid balance status and culture positivity of microbial culture during ICU admission, we hence included these two variables as covariates in the present study [11–13].

### 2.5. Statistical Analyses

Data were represented as means ± standard deviation or number (percentages). We used a Cox proportional hazards model to estimate hazard ratios (HRs) and 95% confidence intervals (CIs) for one-year all-cause mortality after adjustment for potential cofounders. Variables were included in the multivariable model if the associated univariable *P* value was <0.20 and the variance inflation factor was <10. [14] Statistical analyses were two-sided, and the level of significance was set at 0.05. Data analysis was conducted using *R* version 3.6.0.

### 2.6. Sensitivity Analyses

In the present study, we further utilised propensity score matching (PSM) and two weighting methods, including the inverse probability of treatment weight (IPTW) and covariate balancing propensity score (CBPS), to determine the impact of week-one anaemia on the one-year all-cause mortality. [15, 16] In PSM, we used the optimal nearest neighbour matching algorithm, and the calliper distance of standard mean difference was 0.10. The PSM is designed to construct a control group with matched anaemia-associated covariates, but a number of cases were inevitably excluded due to the lack of matched controls. Therefore, the restricted subpopulation might not fully represent the original population. [15] The IPTW, a propensity score weighting method, has been proposed to include the whole population for analyses, but the extreme weight at the tails among the distribution of propensity scores might compromise the balance among covariates [17, 18]. The CBPS, a novel propensity score weighting method, is increasingly used given that CBPS includes the whole study population through weighting and optimises the balance among covariates [16, 19].

## 3. Results

### 3.1. Characteristics of the Enrolled Patients and the Propensity Score-matched Population

**Figure 1:**
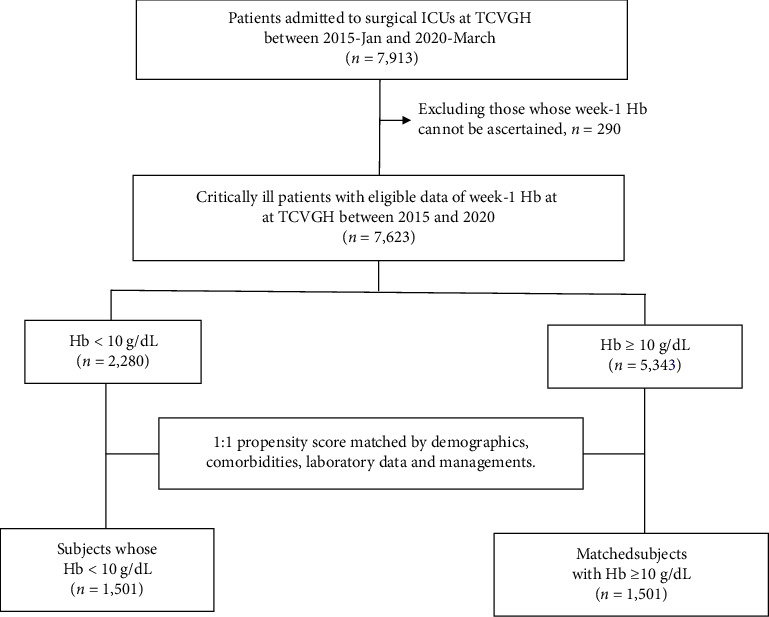
Flowchart of subject enrollment .ICUs, Intensive Care Units; TCVGH, Taichung Veterans General Hospital; Hb, Haemoglobin (g/dL).

Figure 1 shows the process of patient enrollment of the primary cohort (*n* = 7,623) and propensity core-matched cohort (*n* = 3,002) divided by the average level of week-one levels of haemoglobin lower and higher/equal than 10 g/dL (Figure 1). A total of 7,623 patients were included for analyses, and patients with anaemia had a higher proportion of one-year mortality compared with those without anaemia (42.0% vs 17.3%) (Table 1). We found that patients with anaemia had a higher age (64.4 ± 16.3 vs 59.6 ± 15.7 years), slightly lower body mass index (23.8 ± 4.6 vs 24.4 ± 4.5), more comorbidities (CCI 2.0 ± 1.6 vs 1.4 ± 1.3), and more blood loss in the operating room (404.8 ± 612.6 vs 326.2 ± 527.9 mL) compared those without anaemia. The disease severity was higher in those with anaemia, including a higher APACHE II score (22.7 ± 6.6 vs 19.6 ± 5.5) as well as more likely to have shock (50.3% vs 25.3%), to receive emergent surgery (8.0% vs 2.0%), to receive cardiovascular (20.0% vs 15.2%) or major abdominal surgery (14.4% vs 3.2%), to receive mechanical ventilation (77.7% vs 65.5%), to receive red blood cell transfusion (82.9% vs 33.4%), to receive renal replacement therapy (15.9% vs 2.0%), to have a positive fluid balance within day1-3 (1649.9 ± 3010.0 vs 659.7 ± 2062.9 mL) and to have a positive microbiological culture during ICU admission (50.9% vs 26.3%), than those without anaemia. With regards to laboratory data other than haemoglobin, patients with anaemia had a lower platelet count (167.6 ± 99.8 vs 206.6 ± 80.7 10^3^/*μ*L), serum levels of albumin (3.0 ± 0.7 vs 3.7 ± 0.7 mg/dL), and a higher serum levels of creatinine (2.0 ± 2.1 vs 1.0 ± 0.9 mg/dL) compared with those without anaemia. After the PSM, the majority of variables were comparable between the two groups.

### 3.2. Association between Anaemia in the First Week and One-Year Mortality

**Figure 2:**
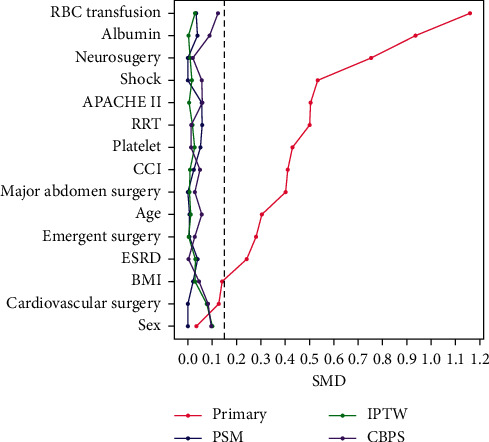


We employed the multivariable Cox proportional hazards model to identify independent one-year mortality predictors in 7,623 critically ill surgical patients. We identified that week-one anaemia (aHR, 1.170; 95% CI, 1.045–1.310) correlated with an increased risk of one-year mortality after adjusting for relevant covariates (Table 2). We then used the propensity score-based approach to further clarify the aforementioned association between week-one anaemia and one-year mortality in critically ill surgical patients.  Figure 2 demonstrates the overall quality of the matching, which was evaluated by comparing the standardised difference of the means as well as the ratio of the variances (Figure 2). The quality of matching was high, with the standardised difference of covariates being lower than 0.10. We adjusted the covariates step-by-step, including demographic and comorbidities in model 2, critical illness relevant disease severity and managements in model 3, and laboratory data in model 4 (Table 3) (See detailed data in supplemental Table 1). We found a consistent strength of association between week-one anaemia and increased risk for one-year mortality in distinct cohorts, with the adjusted HRs in the PSM, IPTW, and CBPS were 1.164 (95% CI 1.025–1.322), 1.179 (95% CI 1.030–1.348), and 1.181 (1.034–1.349), respectively.

## 4. Discussion

In the present study, we used propensity score-matched and -weighted methods to determine the association between week-one anaemia and one-year mortality in critically ill surgical patients. We found that approximately one-third of critically ill surgical patients had anaemia within the first week, and week-one anaemia was associated with a 29.8% increase of the hazard ratio for one-year mortality. The positive association between week-1 anaemia and one-year mortality in critically ill surgical patients was robust in propensity score-based analyses, including PSM, IPTW, and CBPS. Our findings suggest that anaemia appears to be a predictor and modifiable factor for long-term mortality in critically ill surgical patients.

A number of studies have shown the short-term impact of anaemia in critically ill patients, and a few studies have explored the prolonged impact of anaemia after discharge. [2, 20–22] But, few studies have focused on critically ill surgical patients, particularly the long-term mortality impact of anaemia. Shah et al. recently conducted 1,174 ICU patients who were discharged from two mixed medical and surgical ICUs in the United Kingdom (UK) and found that patients discharged from ICU with anaemia (Hb < 10 g/dL) had a longer post-ICU hospital length of stay compared with those in critically patients without anaemia (8 vs 3 days, *p*=0.0017). [3] Indeed, the majority (65.5%, 769/1,174) of enrolled subjects in the aforementioned study were critically ill surgical patients. Similarly, van der Laan et al. analysing 6,358 ICU survivors, mainly discharged from surgical ICU (60.7%, 3860/6358), in Western Australia, found that 45.4% (2,886/6,358) of patients had anaemia (<10 g/dL), which correlated with decreased days at home till day-90 (Relative risk 0.96, 95% CI 0.93–0.98). [4] Notably, anaemia in critically ill patients appears to be a lasting issue. Warner et al. analysed levels of haemoglobin at 3-month, 6-month, and 12-month after ICU discharge among 6,901 critically ill patients in Minnesota and reported that the prevalence of anaemia at 3-month, 6-month, and 12-month were 56%, 52%, and 45%, respectively [21]. Moreover, Warner et al. found that recovery rates from anaemia at 12 months after ICU discharge among patients with mild anaemia, moderate anaemia, and severe anaemia were 58%, 39%, and merely 24%, respectively [21]. These evidence highlight the impacts of anaemia and indicate the need to explore the long-term mortality impact of anaemia in critically ill surgical patients as we have shown in the present study.

The propensity score-based analysis is a statistical approach attempting to reduce selection bias and the confounding effect in an observational study [15]. The propensity score-matching analyses have been widely used, but the matched population may not fully represent the original population [23]. The IPTW overcame the aforementioned issue through weighting the overall enrolled subjects, but extreme propensity scores could bias the estimator and result in excessive variance [18]. Therefore, CBPS is proposed through estimating propensity scores that covariate balance and prediction of treatment assignment are both maximized [16]. The subject number in the present study was high, and standardised mean differences between the two groups among variables were apparently low (Figure 2). Therefore, we identified a similar strength of association between week-one anaemia and one-year mortality among the three propensity score-based analyses.

Anaemia in critically ill surgical patients may result from a wide range of etiologies, consisting of blood loss, persistent/dysregulated inflammation, deficiency of erythropoietin, impaired erythropoietic response, and nutritional deficiencies [2, 24]. Unlike chronic anaemia, inflammation-associated impaired iron metabolism plays a crucial role in the pathogenesis of anaemia among critically ill patients, so-called anaemia of inflammation [25]. Alamo et al. using a rodent model with lung injury and hemorrhagic shock to mimic persistent injury-associated anaemia, demonstrated not only decreased erythropoietin receptor expression despite elevated levels of erythropoietin but also dysregulation of iron homeostasis with decreased levels of plasma hepcidin [26]. Loftus et al. demonstrated that the postinjury inflammation in critically ill trauma patients had higher levels of plasma hepcidin but lower levels of bone marrow erythropoietin and expression of erythropoietin receptor expression [27]. The persistent anaemia among critically ill patients may be attributed to diminished RBC production resulting from nutritional deficiencies, including iron, B12, folate, and other micronutrients [28, 29]. Therefore, the anaemia may at least partly reflect the underlying nutritional status that might affect the recovery from critical illness.

Notably, we think that anaemia should be a modifiable factor in critically ill surgical patients. Accumulating evidence have shown that the administration of iron and erythropoietin cannot improve anaemia or reduce the requirement of blood transfusion in critically ill patients [30, 31]. Moreover, the administration of iron has been implicated with an increased risk of infection [32]. In contrast, patient blood management, particularly the restriction of diagnostic phlebotomy, appears to be practical and actionable in critically ill surgical patients [33]. Helmer et al. recently analysed 38 studies, mainly studies conducted in the United States (14/38, 37%) as well as Canada (5/38, 13%) and the UK (4/38, 10.5%), to explore the diagnostic phlebotomy in critically ill patients and found that the daily phlebotomised blood volume was approximately 40 mL in patients admitted to either medical ICUs or surgical ICUs and those admitted to cardiothoracic ICUs were at higher risk for anaemia than those receiving the noncardiac surgery [33]. In detail, Koch et al. analysed the phlebotomy volume among 1,894 patients who underwent cardiac surgery at Cleveland Clinic in 2012 and reported that the median cumulative volume of blood draws in ICU and hospitalisation were 332 ml and 454 ml, respectively. [34] Koch et al. further found that the majority of blood tests were blood gas (40%), coagulation tests (18%), and complete blood count (14%) [34]. Similarly, Holland J et al. recently quantified the diagnostic blood loss among critically ill surgical patients at St George's Hospital and found the daily average volume drawn was 86.3 ± 19.6 mL [35]. Nevertheless, there is no single criterion used as the indication of RBC transfusion therapy, and accumulating evidence have suggested restrictive transfusion strategies in critically ill patients not only to avoid overtransfusion but also the potential adverse effect of blood transfusion in critically ill patients [36, 37]. These evidences highlight the crucial need for patient blood management and the development of practical blood-sparing strategies, such as restriction of the diagnostic phlebotomy, reduced blood volume collection tubes, early correction of coagulation abnormality, and blood-sparing techniques [38].

There are limitations in this study. First, we cannot claim the causal inference between week-one anaemia and one-year mortality due to the nature of the observational study design, although the consistency and temporality might support causality. Second, multicentre studies are needed to confirm our findings, given that this study is a single-centre study despite the number of subjects number was high in this study. Third, the potential existence of unmeasured confounders, such as iron deficiency status, operative time, and underlying hemoglobinopathy [39], but we have adjusted numerous variables in critical care and used propensity score-based methods to mitigate this concern.

## 5. Conclusions

Anaemia is prevalent comorbidity in critically ill surgical patients, and we linked two databases and employed a propensity score approach to address the long-term mortality impact of anaemia.

We found that approximately one-third of critically ill surgical patients had anaemia within one week, and week-one anaemia correlated with an increased risk of one-year mortality. Our findings highlight the long-term mortality impact of anaemia and shed lights on the essential need of patient blood management program in critically ill surgical patients.

## Figures and Tables

**Table 1:** Characteristics between the patients categorised by week-1 level of haemoglobin in the primary cohort and propensity score-matched cohort.

	Before PSM			1 : 1 PSM		
	Hb < 10	Hb ≥ 10	SMD	Hb < 10	Hb ≥ 10	SMD
	n = 2.280	n = 5.343		n = 1.501	n = 1.501	
Basic characteristics						
Age, years	64.4 ± 16.3	59.6 ± 15.7	0.304	63.3 ± 16.5	63.2 ± 15.6	0.007
Sex (male)	1422 (62.4%)	3423 (64.1%)	0.035	946 (63.0%)	946 (63.0%)	<0.001
Body mass index	23.8 ± 4.6	24.4 ± 4.5	0.140	23.9 ± 4.6	23.8 ± 4.6	0.022
Charlson comorbidity index	2.0 ± 1.6	1.4 ± 1.3	0.410	1.8 ± 1.5	1.8 ± 1.5	0.025

Severity and managements						
APACHE II score	22.7 ± 6.6	19.6 ± 5.9	0.505	21.6 ± 6.4	21.2 ± 6.0	0.057
Presence of shock	1147 (50.3%)	1353 (25.3%)	0.533	663 (44.2%)	663 (44.2%)	<0.001
Emergent surgery	183 (8.0%)	106 (2.0%)	0.280	69 (4.6%)	71 (4.7%)	0.006

Surgical divisions						
Cardiovascular surgery	456 (20.0%)	811 (15.2%)	0.127	386 (25.7%)	386 (25.7%)	<0.001
Neurosurgery	263 (11.5%)	2293 (42.9%)	0.753	246 (16.4%)	246 (16.4%)	<0.001
Major abdomen surgery	328 (14.4%)	172 (3.2%)	0.402	132 (8.8%)	132 (8.8%)	<0.001
Others	495 (21.7%)	558 (10.4%)	0.310	300 (20.0%)	281 (18.7%)	0.032
Blood loss at operating room, ml	404.8 ± 612.6	326.2 ± 527.9	0.137	463.1 ± 633.1	464.5 ± 649.3	0.002
Receiving RBC transfusion	1890 (82.9%)	1785 (33.4%)	1.160	1132 (75.4%)	1110 (74.0%)	0.034
Receiving mechanical ventilation	1771 (77.7%)	3500 (65.5%)	0.272	1133 (75.5%)	1174 (78.2%)	0.065
Receiving RRT	362 (15.9%)	108 (2.0%)	0.500	122 (8.1%)	99 (6.6%)	0.059
End-stage renal disease	92 (4.0%)	25 (0.5%)	0.242	31 (2.1%)	23 (1.5%)	0.040
Fluid balance day 1–3	1649.9 ± 3010.0	659.7 ± 2062.9	0.384	1435.1 ± 2805.6	1159.2 ± 2474.7	0.104
Positive microbiological culture	1161 (50.9%)	1404 (26.3%)	0.523	675 (45.0%)	640 (42.6%)	0.047

Laboratory data						
White blood cell count (10 [3]/*μ*l)	11049.6 ± 5620.9	10792.5 ± 3888.1	0.053	10903.7 ± 5198.4	11147.5 ± 4159.1	0.052
Platelet (10 [3]/*μ*L)	167.6 ± 99.8	206.6 ± 80.7	0.430	173.7 ± 100.2	178.5 ± 87.4	0.052
Albumin (mg/dL)	3.0 ± 0.7	3.7 ± 0.7	0.936	3.2 ± 0.7	3.2 ± 0.7	0.040
Creatinine (mg/dL)	2.0 ± 2.1	1.0 ± 0.9	0.607	1.6 ± 1.8	1.3 ± 1.4	0.236

Outcome						
ICU length of stay, days	12.1 ± 13.5	7.6 ± 10.2	0.377	10.5 ± 12.5	10.4 ± 13.8	0.011
Hospital length of stay, days	29.95 ± 27.8	19.3 ± 23.5	0.409	26.1 ± 23.5	26.6 ± 33.3	0.017
One-year mortality	958 (42.0%)	924 (17.3%)	0.562	535 (35.6%)	442 (29.4%)	0.133

Data are shown as mean ± standard deviation and number (percentages). PSM, propensity score-matching; SMD, standard mean difference; Hb, haemoglobin (g/dL); APACHE, Acute Physiology and Chronic Health Evaluation; RBC, red blood cell; RRT, renal replacement therapy; ICU, intensive care unit.

**Table 2:** Cox proportional hazards regression for one-year mortality among 7,623 critically ill surgical patients.

Characteristics	Univariable		Multivariable	
	HR (95% C.I.)	*p* value	HR (95% C.I.)	*p* value
Basic characteristics				
Age, per 1 year increment	1.026 (1.023–1.029)	<0.001	1.007 (1.004–1.010)	<0.001
Male gender	1.246 (1.131–1.372)	<0.001	1.144 (1.037–1.262)	0.007
Body mass index, per 1 increment	0.946 (0.936–0.957)	<0.001	0.964 (0.953–0.974)	<0.001
**Charlson comorbidity index**	1.308 (1.274–1.343)	<0.001	1.168 (1.134–1.202)	<0.001

**Severity and managements**				
APACHE II score, per 1 increment	1.130 (1.121–1.139)	<0.001	1.058 (1.049–1.068)	<0.001
Presence of shock	2.533 (2.314–2.773)	<0.001	1.508 (1.361–1.671)	<0.001

Surgical divisions				
Cardiovascular surgery	0.366 (0.309–0.435)	<0.001	0.266 (0.221–0.320)	<0.001
Neurosurgery	0.446 (0.398–0.499)	<0.001	0.612 (0.539–0.697)	<0.001
Major abdomen surgery	1.986 (1.722–2.290)	<0.001	0.712 (0.612–0.827)	<0.001
Receiving RBC transfusion	2.452 (2.227–2.700)	<0.001	1.012 (0.894–1.146)	0.851
Receiving mechanical ventilation	1.708 (1.531–1.905)	<0.001	1.093 (0.970–1.231)	0.146
Receiving renal replacement therapy	4.050 (3.574–4.590)	<0.001	1.422 (1.235–1.637)	<0.001
Fluid overload, day 1–3, per 1 litre increment	1.166 (1.149–1.183)	<0.001	1.036 (1.019–1.054)	<0.001
Positive microbiological culture	3.054 (2.787–3.346)	<0.001	1.298 (1.165–1.446)	<0.001

**Laboratory data**				
Platelet (per 10^3^/*μ*L increment)	0.995 (0.994–0.995)	<0.001	0.998 (0.998–0.999)	<0.001
Albumin (per 1 mg/dL increment)	0.434 (0.407–0.464)	<0.001	0.766 (0.709–0.827)	<0.001
Haemoglobin <10 g/dL	2.880 (2.631–3.153)	<0.001	1.170 (1.045–1.310)	0.007

PSM, propensity score matching; IPTW, inverse probability of treatment weighting; CBPS, covariate balancing propensity score; HR, hazard ratio; CI, confidence interval.

**Table 3:** Cox proportional hazard regressions for estimation of the association between level of week-1 haemoglobin lower than 10 g/dL and one-year mortality in critically ill patients.

	PSM	IPTW	CBPS
	HR (95%CI)	HR (95%CI)	HR (95%CI)
**Model 1**	1.264 (1.115–1.434)	1.208 (1.042–1.400)	1.307 (1.140–1.498)

**Model 2**	1.249 (1.101–1.417)	1.184 (1.029–1.362)	1.246 (1.092–1.421)

**Model 3**	1.170 (1.030–1.329)	1.175 (1.026–1.347)	1.186 (1.037–1.356)

**Model 4**	1.164 (1.025–1.322)	1.179 (1.030–1.348)	1.181 (1.034–1.349)

Model 1. Unadjusted. Model 2. Adjusted for demographic data and comorbidities listed in Table 1. Model 3. Adjusted for variables in model 2 and critical illness severity as well as managements listed in Table 1. Model 4. Adjusted for variables in model 3 and laboratory data listed in Table 1. PSM, propensity score matching; IPTW, inverse probability of treatment weighting; CBPS, covariate balancing propensity score; HR, hazard ratio; CI, confidence interval.

## Data Availability

The data underlying this article will be shared upon reasonable request to the corresponding author.

## Supplementary Materials

Supplementary Materials Table 1. Characteristics between the patients categorised by week-1 level of haemoglobin in the IPTW and CBPS cohort.
