# Supplementary material for: Week-One Anaemia was Associated with Increased One-Year Mortality in Critically Ill Surgical Patients
Source: Int J Clin Pract. 2022 Sep 6;2022:8121611. doi: 10.1155/2022/8121611 (PMC9470355; doi:10.1155/2022/8121611)
Supplement: Supplementary Materials — Table 1. Characteristics between the patients categorised by week-1 level of haemoglobin in the IPTW and CBPS cohort. [file 8121611.f1.docx]

| **Table 1. Characteristics between the patients categorised by week-1 level of haemoglobin in the IPTW and CBPS cohort** | | | | | | | | | | | | | | |  |  |  |
| --- | --- | --- | --- | --- | --- | --- | --- | --- | --- | --- | --- | --- | --- | --- | --- | --- | --- |
|  | **IPTW** | | | | | |  | | **CBPS** | | | | | | |  |  |
|  | Hb<10 | | Hb≥10 | | SMD | |  | | Hb<10 | | Hb≥10 | | SMD | | |  |  |
|  | n=7342.7 | | n=7761.6 | |  |  |  | | n=6749.2 | | n=7753.1 | |  |  |  |  |  |
| **Basic characteristics** |  | |  | |  | |  | |  | |  | |  | | |  |  |
| Age, years | 61.3±17.0 | | 61.1±15.7 | | 0.012 | |  | | 61.9±16.9 | | 61.0±15.7 | | 0.057 | | | |  |
| Sex (male) | 4266.8 (58.1%) | | 4892.3 (63.0%) | | 0.101 | |  | | 3954.2 (58.6%) | | 4904.9 (63.3%) | | 0.096 | | | |  |
| Body mass index | 24.0±4.4 | | 24.1±4.5 | | 0.030 | |  | | 24.0±4.4 | | 24.2±4.5 | | 0.046 | | | |  |
| **Charlson comorbidity index** | 1.6±1.5 | | 1.6±1.4 | | 0.009 | |  | | 1.7±1.5 | | 1.6±1.4 | | 0.050 | | | |  |
| **Severity and managements** |  | |  | |  | |  | |  | |  | |  | | | |  |
| APACHE II score | 20.7±6.5 | | 20.7±6.3 | | 0.005 | |  | | 20.9±6.5 | | 20.6±6.3 | | 0.060 | | | |  |
| Presence of shock | 2582.8(35.2%) | | 2669.3(34.4%) | | 0.016 | |  | | 2464.4(36.5%) | | 2617.6(33.8%) | | 0.058 | | | |  |
| Emergent surgery | 328.8(4.5%) | | 342.4(4.4%) | | 0.003 | |  | | 318.6(4.7%) | | 320.9(4.1%) | | 0.028 | | | |  |
| Surgical divisions |  | |  | |  | |  | |  | |  | |  | | | |  |
| Cardiovascular surgery | 1512.1(20.6%) | | 1363.4(17.6%) | | 0.077 | |  | | 1395.4(20.7%) | | 1350.7(17.4%) | | 0.083 | | | |  |
| Neurosugery | 2449.5(33.4%) | | 2554.4(32.9%) | | 0.010 | |  | | 2165.8(32.1%) | | 2561.7(33.0%) | | 0.020 | | | |  |
| Major abdomen surgery | 512.2(7.0%) | | 555.7(7.2%) | | 0.007 | |  | | 508.5(7.5%) | | 526.1(6.8%) | | 0.029 | | | |  |
| Others | 1119.9(15.3%) | | 1015.6 (13.1%) | | 0.062 | |  | | 1047.3(15.5%) | | 1019.5(13.1%) | | 0.068 | | | |  |
| Blood loss at operating room, ml | 417.0±576.1 | | 364.9±578.5 | | 0.090 | |  | | 419.5±582.5 | | 362.4±576.2 | | 0.099 | | | |  |
| Receiving RBC transfusion | | 3719.4(50.7%) | | 3818.7 (49.2%) | | 0.029 | |  | | 3675.1 (54.5%) | | 3743.7 (48.3%) | | 0.124 | | | |
| Receiving mechanical ventilation | | 5089.9(69.3%) | | 5441.2 (70.1%) | | 0.017 | |  | | 4755.3(70.5%) | | 5416.1(69.9%) | | 0.013 | | | |
| Receiving RRT | | 481.2(6.6%) | | 544.7 (7.0%) | | 0.018 | |  | | 477.2(7.1%) | | 521.0(6.7%) | | 0.014 | | | |
| End-stage renal disease | | 121.4(1.7%) | | 160.7 (2.1%) | | 0.031 | |  | | 123.2(1.8%) | | 139.2(1.8%) | | 0.002 | | | |
| Fluid balance day 1-3 | | 898.7±2540.8 | | 1005.6±2489.5 | | 0.043 | |  | | 977.6±2586.1 | | 976.5±2456.3 | | <0.001 | | | |
| Positive microbiological culture | | 2457.1(33.5%) | | 2691.5(34.7%) | | 0.026 | |  | | 2391.4(35.4%) | | 2641.8(34.1%) | | 0.029 | | | |
| **Laboratory data** | |  | |  | |  | |  | |  | |  | |  | | | |
| White blood cell count (10^3^/μl) | 10709.5±5259.4 | | 10964.6±4208.3 | | 0.054 | |  | | 10735.4±5314.8 | | 10954.7±4184.0 | | 0.046 | | | |  |
| Platelet (10^3^/μL) | 195.4±105.0 | | 192.7±84.8 | | 0.028 | |  | | 192.5±104.7 | | 193.7±84.5 | | 0.013 | | | |  |
| Albumin (mg/dL) | 3.5±0.8 | | 3.5±0.8 | | 0.003 | |  | | 3.4±0.8 | | 3.5±0.8 | | 0.089 | | | |  |
| Creatinine (mg/dL) | 1.5±1.7 | | 1.2±1.3 | | 0.205 | |  | | 1.5±1.7 | | 1.2±1.3 | | 0.237 | | | |  |
| **Outcome** |  | |  | |  | |  | |  | |  | |  | | | |  |
| ICU length of stay, days | 8.5±10.5 | | 9.3±13.2 | | 0.069 | |  | | 8.9±10.9 | | 9.2±13.0 | | 0.023 | | | |  |
| Hospital length of stay, days | 22.0±21.7 | | 23.2±30.3 | | 0.044 | |  | | 22.8±22.4 | | 22.9±30.0 | | 0.001 | | | |  |
| One-year mortality | 2159.3(29.4%) | | 1940.3(25.0%) | | 0.099 | |  | | 2064.2 (30.6%) | | 1886.0(24.3%) | | 0.141 | | | |  |
| Data are shown as mean ± standard deviation and number (percentages). Abbreviations: IPTW, inverse probability of treatment weighting; CBPS, covariate balancing propensity score; SMD, standard mean difference; Hb, haemoglobin (g/dL); APACHE, Acute Physiology and Chronic Health Evaluation; RBC, red blood cell; RRT, renal replacement therapy; ICU, intensive care unit. | | | | | | | | | | | | | | | |  |  |
